# Physicians Infrequently Adhere to Hepatitis Vaccination Guidelines for Chronic Liver Disease

**DOI:** 10.1371/journal.pone.0071124

**Published:** 2013-07-26

**Authors:** Kavitha Thudi, Dhiraj Yadav, Kaitlyn Sweeney, Jaideep Behari

**Affiliations:** 1 Division of Gastroenterology, Hepatology, and Nutrition, Department of Medicine, University of Pittsburgh School of Medicine, Pittsburgh, Pennsylvania, United States of America; 2 Department of Basic Sciences, The Commonwealth Medical College, Scranton, Pennsylvania, United States of America; Saint Louis University, United States of America

## Abstract

**Background and Goals:**

Hepatitis A (HAV) and hepatitis B (HBV) vaccination in patients with chronic liver disease is an accepted standard of care. We determined HAV and HBV vaccination rates in a tertiary care referral hepatology clinic and the impact of electronic health record (EHR)-based reminders on adherence to vaccination guidelines.

**Methods:**

We reviewed the records of 705 patients with chronic liver disease referred to our liver clinic in 2008 with at least two follow-up visits during the subsequent year. Demographics, referral source, etiology, and hepatitis serology were recorded. We determined whether eligible patients were offered vaccination and whether patients received vaccination. Barriers to vaccination were determined by a follow-up telephone interview.

**Results:**

HAV and HBV serologic testing prior to referral and at the liver clinic were performed in 14.5% and 17.7%; and 76.7% and 74% patients, respectively. Hepatologists recommended vaccination for HAV in 63% and for HBV in 59.7% of eligible patients. Patient demographics or disease etiology did not influence recommendation rates. Significant variability was observed in vaccination recommendation amongst individual providers (30–98.6%), which did not correlate with the number of patients seen by each physician. Vaccination recommendation rates were not different for Medicare patients with hepatitis C infection for whom a vaccination reminder was automatically generated by the EHR. Most patients who failed to get vaccination after recommendation offered no specific reason for noncompliance; insurance was a barrier in a minority.

**Conclusions:**

Hepatitis vaccination rates were suboptimal even in an academic, sub-speciality setting, with wide-variability in provider adherence to vaccination guidelines.

## Introduction

Hepatitis A and hepatitis B are amongst the most common infectious diseases worldwide [1], [2]. Superinfection with hepatitis A virus (HAV) or hepatitis B virus (HBV) in patients with underlying chronic liver disease is associated with a higher risk of morbidity and mortality [3], [4], [5]. Both HAV and HBV infections are preventable by highly effective and safe vaccines [6], [7], [8], [9]. Experts have recommended screening for susceptibility to HAV and HBV infection and vaccination against them for all patients with chronic liver disease [10], [11]. The CDC and several professional societies have also recommended vaccination against HAV and HBV for susceptible patients with chronic liver disease. In 2008, Centers for Medicare and Medicaid Services (CMS) proposed HAV and HBV vaccination for eligible patients with chronic hepatitis C infection (HCV) as a quality measure [12], [13], [14], [15].

Prior studies have demonstrated shortcomings in adherence to vaccination guidelines in specific subgroups of patients with chronic liver disease in the United States and around the world. In a study of patients with chronic hepatitis C infection in a Veterans Administration Healthy System in California, an adherence rate of 71% for HAV and 70% for HBV and 62% for both vaccination was found [16]. Similarly, in a large cohort of HCV patients from the Department of Veterans Affairs quality measure of HAV and HBV vaccination or documentation of immunity were met in just 57% and 45.5% patients, respectively [17]. A study of patients with autoimmune hepatitis from Germany found vaccination rates of just 11% for HBV and 13% for HAV [18].

Given the increased focus on preventive care and advent of pay for performance models of health care delivery, it likely that adherence to vaccination guideline will be emphasized as a quality measure. Low adherence to vaccination guidelines in primary care settings has been documented [19]. However, limited data are available on whether specialists that care for chronic liver disease patients perform better on these quality measures than community physicians [19]. Furthermore, it is also unknown whether adoption of electronic health records (EHR) and introduction of CMS-mandated quality measures reporting has affected physician practice patterns in terms of adhering to hepatitis vaccination guidelines.

Therefore, the objectives of this study were: (1) To evaluate adherence to hepatitis vaccination guidelines in patients with chronic liver disease at a tertiary care hepatology clinic, (2) to identify barriers to vaccinations in patients with chronic liver disease, and (3) to determine physician variability in adherence to vaccination guidelines.

## Methods

### Study setting

This study was approved by the University of Pittsburgh Medical Center (UPMC) Quality Improvement Committee and the Institutional Review Board waived the need for written informed consent from the participants. The study was conducted at the Center for Liver Diseases (CLD) of the UPMC-Presbyterian Hospital (PUH). PUH is an 800-bed, level 1, fully accredited regional trauma center and the primary teaching hospital of the University of Pittsburgh School of Medicine where residents and fellows train in a wide variety of medical specialties and sub-specialties. The CLD is the outpatient clinic for evaluation and management of patients with liver diseases referred to PUH. The CLD is staffed by 6–8 hepatologists, 3 advanced practice providers, 6–8 nurses, and 8–10 nurse’s aides and medical assistants and 100% of the clinic’s practice is focused on liver diseases.

### Study cohort

We reviewed medical records of all new patients (n = 820) who were evaluated at the UPMC liver clinic in the year 2008 to identify the study population for this study. Patients were eligible if they had chronic liver disease that meets criteria for vaccination for hepatitis A and B, and had a minimum of two follow-up visits during the subsequent 12 months. Patients with chronic hepatitis B (as suggested by positive Hepatitis B surface antigen (HBsAg) were excluded. Therefore, the final study cohort consisted of 705 patients.

### Data Collection

A detailed chart review (including paper records from referring physicians) was performed for all patients who formed the final study cohort to collect information on demographics, referral source, etiology of chronic liver disease, presence of cirrhosis, evidence of decompensation, primary CLD provider, whether serological testing for HAV (hepatitis A antibody [total]) and HBV (HBsAg, hepatitis B surface antibody [HBsAb], hepatitis B core antibody [HBcAb]) was performed (prior to or at the CLD), results of HAV and HBV serology, whether a patient received vaccination prior to CLD referral, whether vaccination was recommended in eligible patients at the CLD, and barriers to vaccination in patients in whom vaccination was recommended at the CLD but who did not get vaccinated. Patients who were recommended but did not receive vaccination were contacted by telephone by one of the investigators (KT) to determine the reason(s). Patients that could not contacted to confirm their vaccination status were categorized as having unknown vaccination status.

In 2008, CMS introduced HAV and HBV vaccination as a PQRI measure for Medicare patients with chronic HCV infection. This PQRI measure was incorporated in the CLD clinical workflow in the form of an electronic reminder window that was activated as soon as the provider chose chronic HCV as a diagnosis for a Medicare patient. Providers were required to complete the PQRI questionnaire before the electronic chart could be closed for billing. To determine whether vaccination recommendation rates differed based on Medicare status of the patients, we electronically retrieved information on whether a patient had Medicare insurance at any point during the year 2008.

### Definition of eligibility for vaccination

For hepatitis A, patients who had a negative HAV antibody were considered eligible for vaccination. For hepatitis B, patients who were negative for HBs antigen, HBs antibody and HBc antibody were considered eligible. Since there are no specific guidelines for vaccination in patients with isolated HBc antibody, we excluded these patients for evaluation of vaccination adherence.

### Statistical analysis

Descriptive analyses are presented as proportions for categorical data, and as median and interquartile range (IQR) for continuous data. Univariate analysis for categorical data was performed using the chi-squared or Fisher’s exact test as appropriate and, for continuous variables, using Mann-Whitney-U test. Data analysis was performed using SPSS software version 19 (SPSS Inc., Chicago, Ill., USA). Two-tailed *p*-values <0.05 were considered significant.

## Results

Table 1 shows data on demographics, referral source, etiology, presence of cirrhosis and insurance status of the 705 patients who formed the final study cohort. About half of the subjects were male, predominantly white and were most frequently referred by either their primary physician or gastroenterologist. Over 80% patients had a single etiology for their chronic liver disease. The most common etiologies were hepatitis C, nonalcoholic fatty liver disease (NAFLD) or nonalcoholic steatohepatitis (NASH) and alcohol. Cirrhosis was present in 39.1% patients and approximately one third of cirrhotics had decompendated disease at the time of evaluation.

**Table 1:** Demographics, etiology and disease status of the study population (N = 705).

Variable	
Age (years) – median (IQR)	53 (44, 60)
Male gender – n (%)	373 (52.9)
Race – n (%)	
White	604 (85.7)
Black	80 (11.3)
Other/Unknown	21 (3.0)
Referral Source – n (%)	
Primary Care Physician	403 (57.2)
Gastroenterologist	116 (16.5)
Other Physicians	75 (10.6)
Unknown	111 (15.7)
Number of etiologies – n (%)	
One	587 (83.3)
Two	111 (15.7)
More than two	7 (1.0)
Etiology – n (%)?	
Hepatitis C	347 (49.2)
Alcohol	122 (17.3)
NAFLD/NASH	199 (28.2)
PBC	33 (4.7)
PSC	29 (4.1)
Autoimmune Hepatitis	46 (6.5)
Hereditary Hemachromatosis	10 (1.4)
Hepatocellular Carcinoma	7 (1.0)
Other Liver tumors	4 (0.6)
Others	33 (4.7)
Cirrhosis – n (%)	276 (39.1)
Decompensated Cirrhosis – n (%)*	99 (35.9)

IQR – interquartile range.

ˆTotal is more than 705 due to overlap of etiologies.

NAFLD – nonalcoholic fatty liver disease; NASH – nonalcoholic steatohepatitis; PBC – Primary biliary cirrhosis; PSC – Primary sclerosing cholangitis.

* Proportion among patients with cirrhosis.

### Testing for Hepatitis A and B status

HAV antibody was tested in 619/705 (87.7%) patients either prior to or at the CLD. Of these, 183/619 (29.5%) tested positive for HAV antibody (Table 2). There were no differences between patients who did and did not have HAV testing based on demographics, referral source, distribution and type of etiology, decompensated cirrhosis and Medicare status.

**Table 2:** Number of new patients with chronic liver disease tested for Hepatitis A and B immunity before or at the liver clinic in the year 2008.

Variable	Before Center for Liver Diseases Visit N (%)	At Center for Liver Diseases N (%)	Before or at Center for Liver Diseases N (%)
Hepatitis A			
Antibody tested	102 (14.5)	541 (76.7)	619 (87.8)
Antibody positive	24 (23.5)	166 (30.6)	183 (29.5)
Hepatitis B			
HBs antigen tested	198 (28.1)	507 (71.9)	637 (90.4)
HBs antibody			
Tested	125 (17.7)	522 (74)	596 (84.5)
Positive	32 (25.6)	154 (29.5)	176 (29.5)
HBc antibody			
Tested	109 (15.5)	524 (74.3)	591 (83.8)
Positive	24 (22.0)	94 (17.9)	111 (18.8)

HAV vaccine received before patient seen at Center for Liver Diseases –47 (6.7).

HBV vaccine received before patient seen at Center for Liver Diseases –67 (9.5).

HBs antigen, HBs antibody and HBc antibody were tested either before or at the CLD in 90.4% (637/705), 84.5% (596/705) and 83.8% (591/705) patients, respectively (Table 2). Of the patients tested, 29.5% were positive for HBs antibody, and 18.8% were positive for HBc antibody. Patients with etiologies other than hepatitis C were more likely to be tested for HBs antigen (92.7% vs. 87.9%, p = 0.03) and those with primary biliary cirrhosis (97% vs. 83.2%, p = 0.03) were more likely to be tested for HBc antibody. Otherwise, there were no differences between patients who did or did not have testing for HBV serology.

### Eligibility for vaccination for Hepatitis A and B at CLD

**Figure 1:**
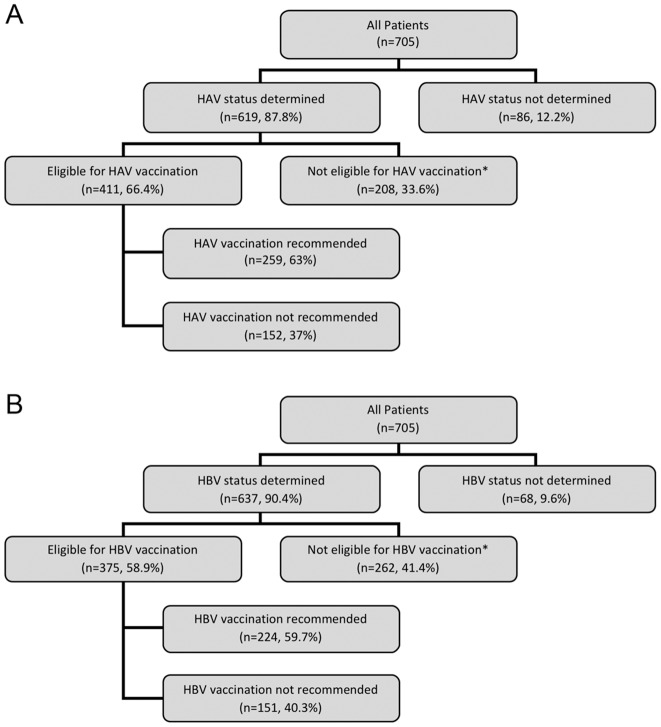
Flowcharts showing how often new patients with chronic liver disease were tested for, eligible for, and were recommended hepatitis vaccination in the liver clinic. A) Hepatitis A vaccination. *Patients not eligible for HAV vaccination included those with positive HAV antibody or who reported completion of the HAV vaccination series. B) HBV vaccination. *Patients not eligible for HBV vaccination included those who had a positive HBs antibody and/or positive HBc antibody or who reported completion of the HBV vaccination series.

After excluding patients who were HAV antibody positive (n = 183) or had received HAV vaccination before referral to the CLD (n = 47; in 25 of these a positive HAV antibody following vaccination was documented), 411 patients were eligible for HAV vaccination at the CLD (Figure 1). After excluding patients who were positive for HBs antibody positive, HBc antibody positive or received HBV vaccination before referral to the CLD (n = 67), 375 patients were eligible for HBV vaccination at CLD (Figure 1b). Patients in whom the serological status for HAV and HBV was unknown (i.e. they did not undergo testing for HAV and/or HBV) were excluded from consideration of eligibility for vaccination.

Patients who were eligible to receive vaccination for HAV and HBV were more likely to be white and have NAFLD/NASH or etiology other than hepatitis C (Tables 3 and 4). While patients eligible to receive HAV vaccination were more likely to be younger, have no cirrhosis and have insurance other than Medicare; those eligible for HBV vaccination were more likely to be older, have cirrhosis and Medicare insurance (Table 3). The proportion of patients who were eligible for vaccination for HAV and HBV were similar across six providers at the CLD (data not shown).

**Table 3:** Eligibility for Hepatitis A vaccination among 619 new chronic liver disease patients and the proportion where it was recommended at the liver clinic.

Variable (N)	Eligible for Vaccination N (%)	p-value	Vaccine Recommended N (%)	p-value
Overall (619)	411 (66.3)	-	259 (63)	-
Age (years) – median (IQR)				
Yes (eligible/recommended)	53 (43, 59)	**<0.001**	53 (42, 60)	0.23
No (not eligible/not recommended)	56 (44, 63)		52 (43, 58)	
Gender				
Male (327)	209 (63.9)	0.17	139 (66.5)	0.15
Female (292)	202 (69.2)		120 (59.4)	
Race				
White (532)	371 (69.7)	**<0.001**	232 (62.5)	0.34
Non White (78)	32 (41)		23 (71.9)	
Hepatitis C				
Yes (298)	174 (58.4)	**<0.001**	104 (59.8)	0.26
No (321)	237 (73.8)		155 (65.4)	
Alcoholic liver disease				
Yes (108)	72 (66.7)	1	43 (59.7)	0.59
No (511)	338 (66.3)		216 (63.7)	
NAFLD/NASH*				
Yes (172)	129 (75)	**0.006**	78 (60.5)	0.51
No (447)	282 (63.1)		181 (64.2)	
Cirrhosis				
Yes (250)	148 (59.2)	**0.002**	95 (64.2)	0.75
No (369)	263 (71.3)		164 (62.4)	
Decompensated cirrhosis				
Yes (87)	53 (60.9)	0.18	28 (52.8)	0.049
No (161)	95 (59)		67 (70.5)	
Number of etiologies				
One (512)	344 (67.2)	0.36	218 (63.4)	0.74
More than one (107)	67 (62.6)		41 (61.2)	
Medicare enrolleê				
Yes (165)	99 (60.0)	**0.043**	68 (68.7)	0.19
No (446)	307 (68.8)		187 (60.9)	

* NAFLD – nonalcoholic fatty liver disease; NASH – nonalcoholic steatohepatitis.

ˆMedicare enrollee at anytime during 2008; information missing in 8 patients.

% for eligible patients is based on the number of overall patients.

% for vaccine recommended is based on the number of patients eligible for vaccination.

**Table 4:** Eligibility for Hepatitis B vaccination among 637 new chronic liver disease patients and the proportion where it was recommended at the liver clinic.

Variable (N)	Eligible for Vaccination N (%)	p-value	Vaccine Recommended N (%)	p-value
Overall (637)	375 (58.9)	-	224 (59.7)	-
Age (years) – median (IQR)				
Yes (eligible/recommended)	55 (46, 63)	**<0.001**	55 (45, 63)	0.56
No (not eligible/not recommended)	52 (40, 58)		54 (46, 61)	
Gender				
Male (338)	192 (56.8)	0.29	116 (60.4)	0.83
Female (299)	183 (61.2)		108 (59.0)	
Race				
White (545)	345 (63.3)	**<0.001**	207 (60.0)	1
Non White (82)	22 (26.8)		13 (59.1)	
Hepatitis C				
Yes (305)	125 (41.0)	**<0.001**	67 (53.6)	0.1
No (332)	250 (75.3)		157 (62.8)	
Alcoholic liver disease				
Yes (111)	74 (66.7)	0.07	38 (51.4)	0.11
No (526)	301 (57.2)		186 (61.8)	
NAFLD/NASH*				
Yes (175)	123 (70.3)	**<0.001**	69 (56.1)	0.37
No (462)	252 (54.5)		155 (61.5)	
Cirrhosis				
Yes (251)	170 (67.7)	**<0.001**	102 (60.0)	1
No (386)	205 (53.1)		122 (59.5)	
Decompensated cirrhosis				
Yes (93)	67 (72)	0.33	35 (52.2)	0.11
No (156)	102 (65.4)		67 (65.7)	
Number of etiologies				
One (532)	310 (58.3)	0.52	190 (61.3)	0.21
More than one (105)	65 (61.9)		34 (52.3)	
Medicare enrolleê				
Yes (165)	110 (66.7)	**0.02**	72 (66.5)	0.16
No (463)	261 (56.4)		149 (57.1)	

* NAFLD – nonalcoholic fatty liver disease; NASH – nonalcoholic steatohepatitis.

ˆMedicare enrollee at anytime during 2008; information missing in 9 patients.

% for eligible patients is based on the number of overall patients.

% for vaccine recommended is based on the number of patients eligible for vaccination.

### Vaccination recommendation for Hepatitis A and B at CLD

In eligible patients, vaccination for HAV and HBV was recommended at the CLD in only 63% and 59.7%, respectively (Tables 3 and 4). While hepatitis A vaccination was recommended more often in eligible patients with no decompensated cirrhosis, the recommendation for HBV vaccination was not dependent on demographics, referral source, distribution and type of etiology, decompensated cirrhosis and Medicare status.

**Figure 2:**
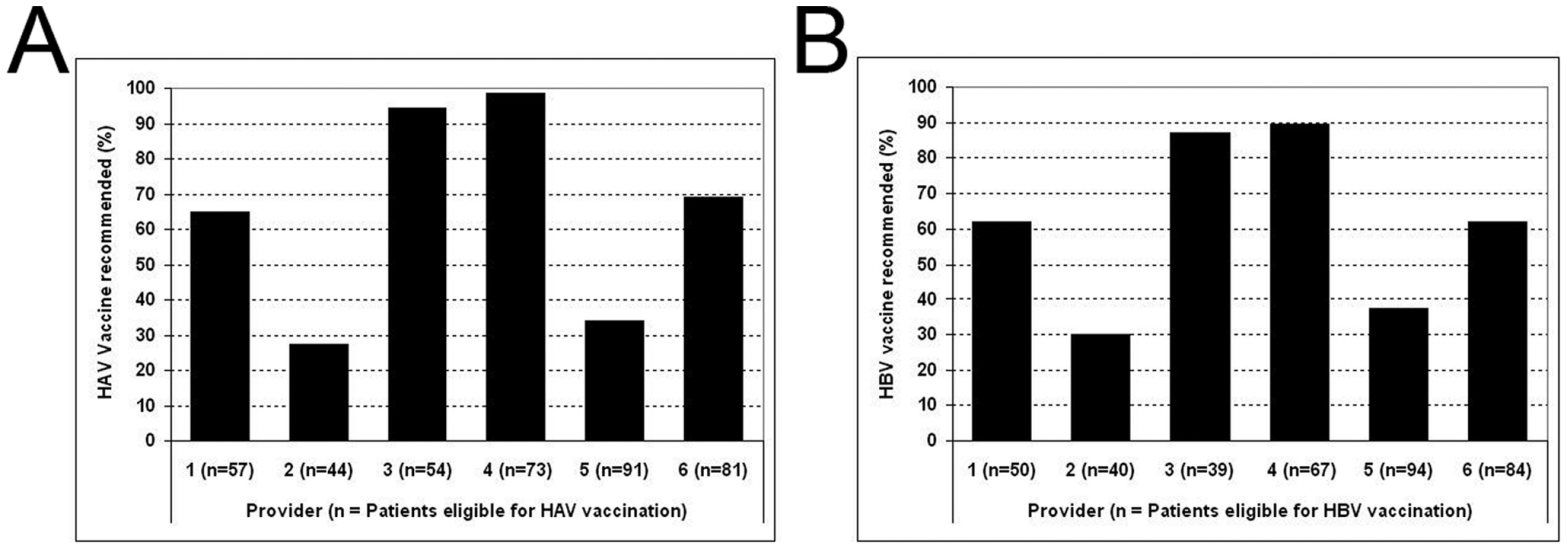
Recommendation for vaccination in eligible patients stratified by liver clinic providers for A) Hepatitis A and B) Hepatitis B.

We found significant variability when we analyzed the data by individual providers (Figure 2). Recommendation for vaccination in eligible patients ranged from 32.4% to 98.6% for hepatitis A and 30% to 89.6% for hepatitis B. When we limited the analyses to patients who had Hepatitis C and Medicare at any point during the study period, the results for vaccination recommendations overall and by provider were similar (data not shown).

#### Barriers to vaccination

Among the 259 patients in whom HAV vaccination was recommended at the CLD, 177 (68.3%) underwent vaccination, 3 (1.2%) had partial vaccination (insurance changed in 1, lost in 1 and no specific reason in 1), 38 (14.7%) did not undergo vaccination, and the status of vaccination was unknown in the remaining 41 (15.8%) patients. The reasons for not getting vaccination (n = 38) were: no clear reason (24), were on other medications (hydroxychloroquine and infliximab in one each), PCP’s office did not have vaccines or did not give it (4), no prescription given or did not know where to get the vaccine (3), no insurance coverage (3), lost script (1), and did not have PCP (1).

## Discussion

Our study makes several important contributions to the literature. First, we demonstrate that adherence to HAV and HBV vaccination guidelines in a specialty hepatology clinic, while higher than rates reported in community-based practices, is far from optimal. Second, we show that significant variability in adherence to vaccination guidelines exists amongst providers within the same practice. Third, we find that an EHR-based electronic reminder system did not improve vaccination recommendation rates. Finally, we find that insurance is not a major impediment to vaccination but lack of adequate counseling and patient education may be a factor in patient compliance with vaccination recommendations.

In the current healthcare climate, there is increased focus on preventive care as a key intervention to provide the most cost-effective care [20]. We found that just 30% of patients were immune to HAV and HBV infection prior to referral to the liver clinic. Our results are similar to a previous study, which reported that vaccination recommendation rates in primary care clinics were lower than in specialty clinics [19]. Thus, increasing awareness of hepatitis vaccination among primary care physicians is another strategy to potentially improve vaccination rates in patients with chronic liver disease. Surprisingly, we found that insurance was a barrier to vaccination in only a small number of patients [21]. We speculate that inadequate explanation or counseling for the need for vaccination may be one potential source for noncompliance with vaccination recommendation. It is likely that this noncompliance may be amenable to approaches such as patient educational material or involvement of nursing staff or advanced practitioners in the process.

Recent studies have highlighted variations in quantifiable outcomes and adherence to well-established guidelines amongst primary care physicians as well as specialists [22], [23], [24], [25]. Given that the demographic, etiological, and clinical characteristics of patients were similar between the providers, the observed variability in vaccination recommendations cannot be explained by these patient-specific factors. We did not measure the characteristics of individual physicians that correlated with adherence to vaccination guidelines but further research is warranted to elucidate these variables.

In our clinic, a “pop-up” electronic reminder was activated for every Medicare patients with a billing diagnosis of HCV infection and providers could not sign-off on these charts without addressing specific questions about these patients' hepatitis vaccination status. While there was a small financial incentive associated with complying with the PQRI queries, there was no penalty associated with noncompliance. We found no difference in vaccine recommendation rates between this cohort of Medicare patients and the rest of the patient population. Future prospective studies will be needed to address the question whether such EHR-based reminder systems can improve compliance with vaccination guidelines.

Several limitations of this study should be noted. First, this was a retrospective study that was performed at a single center. Second, we did not record characteristics of individual physicians or practice pattern differences between physicians and their advanced practice providers that might explain the individual variability in vaccination recommendation rates. Third, since EHR-based reminders were only required during the study period for Medicare patients with hepatitis C infection, our results on automated computerized reminders may not be generalizable to other etiologies and groups of patients.

In summary, we show here that adherence to hepatitis vaccination guidelines, while higher than reported in community-based clinics, was low even in an academic hospital-based hepatology clinic. Furthermore, there were significant variations amongst providers, which did not correlate with the number of patients seen by individual physicians, within the same practice in adhering to vaccination guidelines in eligible patients. Finally, most patients who were recommended vaccination but failed to get them offered no specific reason for their noncompliance, highlighting the need for better education and counseling of patients with regard to this important preventive intervention.

## References

[1] Ott JJ, Stevens GA, Groeger J, Wiersma ST (2012) Global epidemiology of hepatitis B virus infection: new estimates of age-specific HBsAg seroprevalence and endemicity. Vaccine 30: 2212–2219. 22273662 10.1016/j.vaccine.2011.12.116

[2] Wasley A, Fiore A, Bell BP (2006) Hepatitis A in the era of vaccination. Epidemiologic reviews 28: 101–111. 16775039 10.1093/epirev/mxj012

[3] Almasio PL, Amoroso P (2003) HAV infection in chronic liver disease: a rationale for vaccination. Vaccine 21: 2238–2241. 12744849 10.1016/s0264-410x(03)00139-7

[4] Keeffe EB (1995) Is hepatitis A more severe in patients with chronic hepatitis B and other chronic liver diseases? The American journal of gastroenterology 90: 201–205. 7847285

[5] Liaw YF, Yeh CT, Tsai SL (2000) Impact of acute hepatitis B virus superinfection on chronic hepatitis C virus infection. The American journal of gastroenterology 95: 2978–2980. 11051381 10.1111/j.1572-0241.2000.02337.x

[6] Andre F, Van Damme P, Safary A, Banatvala J (2002) Inactivated hepatitis A vaccine: immunogenicity, efficacy, safety and review of official recommendations for use. Expert review of vaccines 1: 9–23. 12908508 10.1586/14760584.1.1.9

[7] Innis BL, Snitbhan R, Kunasol P, Laorakpongse T, Poopatanakool W, et al (1994) Protection against hepatitis A by an inactivated vaccine. JAMA : the journal of the American Medical Association 271: 1328–1334. 8158817

[8] Szmuness W, Stevens CE, Harley EJ, Zang EA, Oleszko WR, et al (1980) Hepatitis B vaccine: demonstration of efficacy in a controlled clinical trial in a high-risk population in the United States. The New England journal of medicine 303: 833–841. 6997738 10.1056/NEJM198010093031501

[9] Zuckerman JN (2006) Protective efficacy, immunotherapeutic potential, and safety of hepatitis B vaccines. Journal of medical virology 78: 169–177. 16372285 10.1002/jmv.20524

[10] Keeffe EB (2005) Acute hepatitis A and B in patients with chronic liver disease: prevention through vaccination. The American Journal of Medicine 118 Suppl 10A21S–27S. 10.1016/j.amjmed.2005.07.013 16271537

[11] Lau DT, Hewlett AT (2005) Screening for hepatitis A and B antibodies in patients with chronic liver disease. The American Journal of Medicine 118 Suppl 10A28S–33S. 10.1016/j.amjmed.2005.07.014 16271538

[12] Fiore AE, Wasley A, Bell BP (2006) Prevention of hepatitis A through active or passive immunization: recommendations of the Advisory Committee on Immunization Practices (ACIP). MMWR Recommendations and reports : Morbidity and mortality weekly report Recommendations and reports/Centers for Disease Control 55: 1–23. 16708058

[13] Mast EE, Margolis HS, Fiore AE, Brink EW, Goldstein ST, et al (2005) A comprehensive immunization strategy to eliminate transmission of hepatitis B virus infection in the United States: recommendations of the Advisory Committee on Immunization Practices (ACIP) part 1: immunization of infants, children, and adolescents. MMWR Recommendations and reports: Morbidity and mortality weekly report Recommendations and reports/Centers for Disease Control 54: 1–31. 16371945

[14] Mast EE, Weinbaum CM, Fiore AE, Alter MJ, Bell BP, et al. (2006) A comprehensive immunization strategy to eliminate transmission of hepatitis B virus infection in the United States: recommendations of the Advisory Committee on Immunization Practices (ACIP) Part II: immunization of adults. MMWR Recommendations and reports: Morbidity and mortality weekly report Recommendations and reports/Centers for Disease Control 55: 1–33; quiz CE31–34. 17159833

[15] Pham H, Geraci SA, Burton MJ (2011) Adult immunizations: update on recommendations. The American journal of medicine 124: 698–701. 21658665 10.1016/j.amjmed.2010.07.032

[16] Hernandez B, Hasson NK, Cheung R (2009) Hepatitis C performance measure on hepatitis A and B vaccination: missed opportunities? The American journal of gastroenterology 104: 1961–1967. 19491840 10.1038/ajg.2009.252

[17] Kramer JR, Hachem CY, Kanwal F, Mei M, El-Serag HB (2011) Meeting vaccination quality measures for hepatitis A and B virus in patients with chronic hepatitis C infection. Hepatology 53: 42–52. 21254161 10.1002/hep.24024

[18] Worns MA, Teufel A, Kanzler S, Shrestha A, Victor A, et al (2008) Incidence of HAV and HBV infections and vaccination rates in patients with autoimmune liver diseases. The American journal of gastroenterology 103: 138–146. 17970833 10.1111/j.1572-0241.2007.01609.x

[19] Jacobs RJ, Meyerhoff AS, Saab S (2005) Immunization needs of chronic liver disease patients seen in primary care versus specialist settings. Digestive diseases and sciences 50: 1525–1531. 16110847 10.1007/s10620-005-2873-5

[20] Woolf SH (2008) The power of prevention and what it requires. JAMA: the journal of the American Medical Association 299: 2437–2439. 18505953 10.1001/jama.299.20.2437

[21] Keeffe EB (2006) Hepatitis A and B superimposed on chronic liver disease: vaccine-preventable diseases. Transactions of the American Clinical and Climatological Association 117: 227–237; discussion 237–228. PMC1500906 18528476

[22] Barclay RL, Vicari JJ, Doughty AS, Johanson JF, Greenlaw RL (2006) Colonoscopic withdrawal times and adenoma detection during screening colonoscopy. The New England journal of medicine 355: 2533–2541. 17167136 10.1056/NEJMoa055498

[23] Chamie K, Saigal CS, Lai J, Hanley JM, Setodji CM, et al (2011) Compliance with guidelines for patients with bladder cancer: variation in the delivery of care. Cancer 117: 5392–5401. 21780079 10.1002/cncr.26198 PMC3206145

[24] Prevedello LM, Farkas C, Ip IK, Cohen AB, Mukundan S, et al (2012) Large-scale automated assessment of radiologist adherence to the Physician Quality Reporting System for stroke. Journal of the American College of Radiology: JACR 9: 414–420. 22632668 10.1016/j.jacr.2012.01.014

[25] Sherman BW, Sekili A, Prakash ST, Rausch CA (2011) Physician-specific variation in medication adherence among diabetes patients. The American journal of managed care 17: 729–736. 22084892

